# Determinants of Acute Asthma Attack among adult asthmatic patients visiting hospitals of Tigray, Ethiopia, 2019: case control study

**DOI:** 10.1186/s40733-020-00054-w

**Published:** 2020-04-03

**Authors:** Melaku Negash, Hagos Tsegabrhan, Teklit Meles, Degena Bahrey Tadesse, Gebreamlak Gidey, Yemane Berhane, Kibrom Berhanu, Tsgalem Haylemaryam

**Affiliations:** 1 Department of adult health nursing ,school of Nursing, Aksum University, Aksum, Ethiopia; 2 Department of Psychiatric, Mekelle University, Mekelle, Ethiopia; 3 Adwa General Hospital, Adwa, Ethiopia; 4 Department of midwifery, Aksum University, Aksum, Ethiopia; 5 college of medicine and health science, Adigrat university, Adigrat, Ethiopia; 6 Maternity and reproductive health nursing, Mekelle University, Mekelle, Ethiopia; 7 Department of Emergency and critical care nursing, Mekelle University, Mekelle, Ethiopia

**Keywords:** Acute asthma attack, Adult, Determinants, Ethiopia

## Abstract

**Introduction:**

Acute asthma attack is one of the most common causes of visits to hospital emergency departments in all age groups of the population and accounts for the greater part of healthcare burden from the disease. Despite, Acute asthma attack is an important public health problem that affects not only the patients, but also to the family, health professionals, health care institutions and development of the nation, little is known about the risk factors of acute asthma attack.

Therefore, this study is aimed to investigate the determinants of acute asthma attack among.

**Objective:**

The aim of this study was to assess the determinant factors of acute asthma attack among adult asthmatic patients visiting general hospitals of central zone, Tigray, Ethiopia, 2019.

**Method:**

Hospital based unmatched case control study design was conducted in general hospitals of central zone of Tigray, Ethiopia 2019. Data were collected using pretested interviewer administered questionnaire. A total of 289 study subjects (96 cases &193 controls) were selected by systematic random sampling. Data were entered to Epi data version 3.1 then exported to SPSS version 23 for analysis. Bivariate logistic regression was employed to examine the statistical association between dependent and independent variables. Variables with *p* value < 0.25 in binary logistic regression were entered to multivariable logistic regression model and variables with p value < 0.05 was taken as significant determinants of the outcome variable.

**Result:**

A total of 96 adult asthmatic patients who have acute asthma attack (cases) and 193 adult asthmatic patients without attack (controls)) with 100% response rate were participated in this study. Upper Respiratory tract Infection [AOR = 6.835,95% CI = 3.285,14.222], Season [AOR =2.204,95% CI = 1.011,4.805] kitchen smoke [AOR = 2.307,95%CI1.010,5.272]& sleep apnea [AOR = 9.254, 5%CI =3.563,25.460] were significantly associated with acute asthma exacerbation.

## Introduction

Asthma is a long-term inflammatory disease of the respiratory system which is characterized by wheezing, shortness of breath, chest tightness. Globally it affects approximately 300 million people and is estimated to rise to 400 million by 2025 globally [1, 2]. And it is ranked 16th among the leading causes of disability and 28th among the leading causes of burden of disease, as measured by disability adjusted life years (DALYs) [3].

According to Croatian medical journal 2013, an estimate of asthma prevalence in Africa, was 49.7 million in the age of < 15 years (13.9%), < 45 years 102.9 million (13.8%), and in total population 119.3 million (12.8%) in 2010 [4].

Asthma exacerbation is defined as a worsening of shortness of breath, cough, wheezing, or chest tightness. If not treated immediately there will be increase in flow resistance causing increased work of breathing, gas exchange inefficiency, respiratory muscle tiredness and finally hypercapnic and hypoxemic respiratory failure [5]. This implies that acute asthma attack is a significant public health problem that affects patients with their parents or families and the community through labor and school loss, frequent emergency clinic visits, a poor quality of life hospitalizations and finally death [6]. According to Centers for Disease Control and prevention (CDC) report, More than 11 million people reported having an acute asthma attack [7].

Despite, in Ethiopia little is known about how risk factors are associated with exacerbation, according to asthma severity and the relative importance of the risk factors. This may be the reason for no policy and strategy to ascertain and acting out of effective intervention in order to reduce the burden of acute asthma attack [8]. Therefore, this study is aimed to full fill this gap.

## Methods

### Study setting and study design

Hospital based unmatched case control study was conducted in the selected general Hospitals of Central zone of Tigray from November 2018 to July 2019.

### Study population and sample size determination

#### Source population

##### Cases

All adult asthmatic patients visited to emergency unit who have acute asthma attack.

##### Control

All adult patients diagnosed as asthma but without acute asthmatic attack who visited the OPD and the regular follow-up unit during the data collection period.

#### Study population

##### Cases

All selected adult asthmatic patients visited to emergency unit who have acute asthma attack during the data collection period.

##### Control

All selected adult patients diagnosed as asthma but without acute asthmatic attack who visited the OPD and the regular follow-up unit during the data collection period.

### Eligibility criteria

#### Inclusion criteria

##### Cases

Adult asthmatic patients who have acute asthma attack during the data collection period.

##### Controls

Adult asthmatic patient without acute asthma attack during the data collection period.

#### Exclusion criteria

Patients with any history of pulmonary embolism, chronic obstructive pulmonary disease, active pulmonary TB, known congestive heart failure and known mechanical obstruction.

### Sample size determination

Sample size was calculated from Previous study conducted in Uganda [9],using Epi info version 7. sample size was determined based on the assumption of confidence level = 95%; Power = 80%; Odds ratio = 2.132 with case to control ratio = 1:2, proportion of among controls 37.2%, proportion of among cases = 55.8%.

Therefore, the required sample size for cases was =92 where as for the controls =183 and the overall sample size was = 275 then after adding 5% non-response rate, the total sample size was 289. Finally, a sample size for cases was 96 and for controls 193.

### Sampling technique and procedure

The total sample size was allocated to each hospital proportionally based on the number of patients who attend in the selected hospitals. A total number of 585(case 165, control.420) patients attended at the selected Hospitals with in 2 months of the previous year (April 1 to May 30–2018). Systematic random sampling method was applied in each hospital to select 289 participants.

### Study Variables

#### Dependent Variable

Acute asthma attack.

#### Independent variables

##### Socio-demographic variables

Age, Gender, Marital status, Residence, Educational level, Employment status and Occupational status.

##### Behavioral factors

Exercise, vigorous activity Smoking cigarette.

##### Environmental factors

Humidity, Kitchen smoke, dust, Season.

##### Medical and Clinical characteristics

URTI, Sleep apnea, Missing follow-up / appointments,

### Operational definitions

#### Asthma

Those who present with cough, wheezing and difficulty of breathing and diagnosed asthma by physician [10].

#### Acute Asthma Attack

Those who present with worsening of wheezing, shortness of breath, cough, chest tightness and diagnosed as acute asthma attack by physician [10].

**Smoker:(**daily smoker and non-daily smoker) those who currently smokes or those who quit smoking less than 1 year before the assessment [10].

**Passive smoker:** Smoke inhaled involuntarily by non-smokers [11].

**Nonsmoker:** Respondents who report never smoke those who quit smoking greater than 1 year before the assessment.

**Vigorous activity:** participants doing activity more than 10 min continuously, that increases breathing, like carrying or lifting heavy loads, digging or construction work, cutting fire wood [11].

### Data collection tool

Structured questionnaire was used to collect the data which was adapted from different literatures [9, 12–14]. The questionnaire contains four parts: socio-demographic, environmental factors, behavioral factors, and Medical &Clinical characteristics.

### Data collection procedures

Data were collected from cases and controls using structured questionnaire and checklists through face-to-face interview and from patients chart review respectively.

Twelve BSc nurses as data collectors and three senior nurse supervisors were recruited for the data collection, Then data from cases were collected after they take all the necessary medical care and they recover from their attack whereas from the controls data were collected after they have completed their assessment by physician and at the last record reviews from their chart. Participants were identified as having upper respiratory tract infection and Obstructive sleep apnea from their medical charts which was diagnosed by senior physicians. This is to mean that, it was just suspected clinically by the time of the acute event. The reason we obeyed to use clinically diagnosis for obstructive sleep apnea is that, there is no accesses of modern diagnostic modality like polysomnography in the study area which was Tigray regional state not only in the study area but also in the country Ethiopia as a whole. The evaluation protocol that we use were a single evaluation visit for each case and even those who have follow-up and developed acute asthma attack were included*.*

### Data quality control techniques

Data quality was ensured by training of data collectors and supervisors before data collection period. 5% of the questionnaire was pre-tested in Shire Hospital which was not included in the actual data collection. Based on the findings of the pre-test, questionnaire was modified. The filled questionnaire was checked for completeness and accuracy by data collectors, supervisors and principal investigator each day.. The questionnaire was translated into Tigrigna language for better understanding to both the data collectors and respondents and then back translated into English by another expert to ensure accuracy and consistency.

### Data analysis procedures

Data were entered in to Epi data version 3.1 and analyzed using SPSS version 23.0. The degree of association between independent and dependent variables were assessed using adjusted odds ratio with 95% confidence interval. Variables < 0.25 *p*-value in binary logistic regression were entered to multivariable logistic regression model to control the potential confounding variables. Variables with p-value less than 0.05 in multivariable logistic regression model were taken as significantly associated factors. Variance inflation factor (VIF) was used to assess Multicollinearity between the independent variables. Hosmer and Lemeshow goodness fit model were used to check model fitness.

#### Ethical consideration

Ethical clearance was obtained from Mekelle University College of health sciences institutional review board (IRB). A subsequent permission was also obtained from Tigray teaching hospitals. Respondents were informed about the purpose of the study and the interview was conducted after receiving the written consent from participants. Confidentiality of the data/information was secured and was not used for other purposes.

## Results

### Sociodemographic characteristic of study participants

Among the participants, 67.7% (65) of the cases and 60.6% (117) of the controls were females. The median ages of participants were 43 years with interquartile range (IQR) of 26.5 years among cases and 43 median ages with interquartile range (IQR) of 22 for control.

The educational status, one third 33.3% (32) of the cases and 24.9% (48) of the controls were collage and above, where as 14.6% (14) of the cases and 16.6% (32) of the controls were unable to read and write. The majority of the cases 63.5% (61) and 60.1% (116) of the controls were married (Table 1).

**Table 1:** Sociodemographic characteristics of adult asthmatic patients visiting general hospitals of central zone, Tigray, Ethiopia, 2019

Variables		Cases, n (%)	control n (%)	Total,n (%)
Sex	Male	31(32.3)	76(39.4)	107(37)
	Female	65(67.7)	117(60.6)	182(63)
Age group	18–25	12(12.5)	21(10.9)	33(11.4)
	26–35	20(20.8)	40(20.7)	60(20.8)
	36–45	20(20.8)	43(22.3)	63(21.8)
	46–60	28(29.2)	61(31.6)	89(30.8)
	+ 60	16(16.7)	28(14.5)	44(15.2)
Religion	Orthodox	86(89.6)	174(90.2)	260(90)
	Muslim	10(10.4)	19(9.8)	29(10)
Ethnicity	Tigrian	90(93.8)	184(95.3)	274(94.8)
	Other	6(6.2)	9(4.7)	15(5.2)
Education	Unable to read & write	14(14.6)	32(16.6)	46(15.9)
	Able to read & write	12(12.5)	52(26.9)	64(22.1)
	Primary school	15(15.6)	26(13.5)	41(14.2)
	Secondary school	23(24)	35(18.1)	58(20.1)
	Collage and above	32(33.3)	48(24.9)	80(27.7)
Occupational status	Governmental	39(40.6)	73(37.8)	112(38.8)
	Self-employed	35(36.5)	61(31.6)	96(33.2)
	Unemployed	22(22.9)	59(30.6)	81(28)
Marital status	Single	21(21.9)	48(24.9)	69(23.9)
	Married	61(63.5)	116(60.1)	177(61.2)
	Divorced	6(6.3)	14(7.3)	20(69)
	Widowed	8(8.3)	15(7.8)	23(8)
Residence	Urban	57(59.4)	121(62.7)	178(61.6)
	Rural	39(40.6)	72(37.3)	111(38.4)
Income	**<** 500	13(13.5)	38(19.7)	51(17.6)
	501–1500	23(24)	45(23.3)	68(23.5)
	1501–2500	15(15.6)	21(10.9)	36(12.5)
	> 2500	45(46.9)	89(46.1)	134(46.4)

### Behavioral characteristics of study participants

Among the participants, 2.1% (2) of the cases and 1.1% (6) of the controls were smokers.in parallel with this 3.1% of the cases and 4.7% of the control were passive smokers. Regarding vigorous activity 37.5% (36) of the cases and 23.8% (46) of the controls were do vigorous activity. Majority of the participants 72.9% (70) of the cases and 58% (112) of the controls were doing exercise.

### Medical & clinical characteristics of study participants

Among the participants, 44.8% (43) of the cases and 13.5% (26) of the controls had Upper Respiratory Tract Infections (URTI) and 29.2% (28) of the cases and few of the controls 5.2% (10) had obstructive sleep apnea.

Among the participants, 31.3% (30) of the cases and 20.7% (40) of the controls had Missing follow up.

### Environmental characteristics of study participants

Regarding the seasons of a year, spring season (April, May, June) were the season with high percentage 37.7% (109) of acute asthma attack than the autumn season. Majority of the participants 79.5% (230) were open their window/door while they were cooking. Concerning the kitchen of the participants 32.3% (31) of the cases and 20.2% (39) of the control’s kitchen have no kitchen smoke (chimney) (Table 2).

**Table 2:** Environmental characteristics of asthmatic patients visiting general hospitals of central zone, Tigray, Ethiopia, 2019

Variables		Cases, n (%)	control n (%)	Total, n (%)
Season	Autumn	27(28.1)	62(32.1)	89(30.8)
	Winter	10(10.4)	28(14.5)	38(13.1)
	Spring	48(50)	61(31.6)	109(37.7)
	Summer	11(11.5)	42(21.8)	53(18.3)
window /door while cooking	yes	76(79.2)	163(84.5)	230(79.5)
	No	20(20.8)	30(15.5)	59(20.5)
Kitchen smoke	Yes	65(67.7)	154(79.8)	219(75.8)
	No	31(32.3)	39(20.2)	70(24.2)
Exposed to vapor	Yes	6.3(6)	11(5.7)	17(5.9)
	No	93.7(90)	182(94.3)	272(94.1)
Exposed to gas	Yes	23(24)	43(22.3)	66(22.8)
	No	73(76)	150(77.7)	223(77.2)
Exposed to dust	Yes	87(90.6)	159(82.4)	246(85.1)
	No	9(9.4)	34(17.6)	43(14.9)
Exposed to fume	Yes	40(41.7)	93(48.2)	133(46)
	No	56(58.3)	100(51.8)	156(54)
Exposed to Humidity	Yes	33(34.4)	64(33.2)	97(33.6)
	No	63(65.6)	129(66.8)	192(66.4)
Cooking	Wood	33(34.4)	66(34.2)	99(34.3)
	Gas	8(8.3)	15(7.8)	23(8)
	Electric	55(57.3)	112(58)	167(57.8)

**Table 3:** Bivariate and multivariable logistic regression analyses of acute asthma attack among adult asthmatic patients visiting general hospitals of central zone, Tigray, Ethiopia, 2019

Variables		Cases n (%)	Control n (%)	Crude OR	Adjusted OR	***P***-value
Education Status	No read& write		14(14.6)	32(16.6)	1	
	read & write	12(12.5)	52(26.9)	0.527(0.217,1.282)	0.690 (0.214,2.225)	0.535
	Primary school	15(15.6)	26(13.5)	1.319(0.540,3.222)	0.730 (0.213,2.503)	0.617
	Secondary school	23(24)	35(18.1)	1.502(0.662,3.408)	1.539 (0.520,4.558)	0.436
	Collage & above	32(33.3)	48(24.9)	1.524(0.705,3.295)	2.181 (0.776,6.129)	0.139
Vigorous Activity	Yes	36(37.5)	46(23.8)	1.917(1.129,3.256)	1.733 (0.825,3.639)	0.147
	No	60(62.5)	147(76.2)	1	1	
Exercise	Yes	70(72.9)	112(58)	1.947(1.142,3.319)	1.171(0.577,2.377)	0.663
	No	26(27.1)	81(42)	1	1	
Season	Autumn	27(28.1)	62(32.1)	1	1	
	Winter	10(10.4)	28(14.5)	0.820(0.350,1.922)	0.900 (0.317,2.557)	0.843
	Spring	48(50)	61(31.6)	1.807(1.002,3.257)	2.204(1.011,4.805)	0.047**
	Summer	11(11.5)	42(21.8)	0.601(0.269,1.343)	0.599(0.217,1.653)	0.322
Exposed to dust	Yes	87(90.6)	159(82.4)	2.067(0.948,4.509)	2.702 (0.923,7.912)	0.070
	No	9(9.4)	34(17.6)	1	1	
Window	Yes	76(79.2)	163(84.5)	1	1	
	No	20(20.8)	30(15.5)	1.556(0.824,2.935)	1.384(0.573,3.344)	0.470
Kitchen smoke	Yes	65(67.7)	154(79.8)	1	1	
	No	31(32.3)	39(20.2)	1.883(1.083,3.276)	2.307(1.010,5.2725)	0.047**
URTI	Yes	43(44.8)	26(13,5)	5.211(2.927,9.277	6.835(3.285,14.222)	<.001**
	No	53(55.2)	167(86.5)	1	1	
Sleep apnea	Yes	28(29.2)	10(5.2)	7.535(3.476,16.337)	9.524(3.563,25.460)	<.001**
	No	68(70.8)	183(94.8)	1		
Missing follow up	Yes	30(31.3)	40(20.7)	1.739(0.999,3.027)	1.946(0.929,4.075)	0.078
	No	66(68.8)	153(79.3)	1	1	

Key: * Indicates risk factors for asthma exacerbations in bivariate analysis.

** Indicates factors associated with asthma exacerbation at multivariate analysis

## Discussion

Unmatched case control study with 96 cases and 193 controls was conducted to show the determinants of acute asthma attack among adult asthmatic patients visiting general hospitals of central zone, Tigray, Ethiopia.

Having URTI increases the occurrence of acute asthma attack 6.8 times [AOR = 6.835,95% CI = 3.285,14.222] than those who have not upper respiratory tract infection (URTI) (Table 3).

This is consistent with the studies conducted in Gondar, Uganda and Ireland [9, 12, 15].

The association might be due to the mechanism of airway inflammation,mucus hyper secretion, and bronchial hyper responsiveness [16]. In contrast to this study upper respiratory tract infections was no risk factor for acute asthma exacerbation on the study conduct in Pretoria and New Zealand [14, 17]. This difference might be due to difference in health care seeking behavior of the participants in this study.

This study revealed that, sleep apnea was strongly associated with the occurrence of acute asthma exacerbation. Those who have sleep apnea are 9.5 times more likely to run in to acute asthma exacerbation than those who have not sleep apnea [AOR = 9.524, 95% CI = 3.563, 25.460].

This findings is comparable with a study done in Gondar and USA [12, 18].

The possible reason is the fact that sleep apnea lead to the worsening of asthma control in patients with concomitant sleep apnea secondary to bronchoconstriction as a result of increase vagal tone while sleeping [19].

The result of this study shows that the odds of having acute asthma in Spring season was 2.2 times higher than the odds of having acute asthma attack in the autumn season [AOR = 2.204,95% CI = 1.011,4.805]. This is consistent with a study conducted in Canada in which spring season was triggering factor for asthma exacerbation [20]. Seasonal variation is the risk factors for acute asthma attack especially pollens appearing seasons like spring season exacerbates acute asthma attack. This may be due to the reason that during the spring, tree pollen, mold spores and grass have the power to inflame and narrow the air passages of people who have asthma [21].

The result of this study was different from a study conducted in Spain which was resulting winter season as higher risk of developing acute asthma attack [22]. The difference could be arisen from seasonal variation between the study areas, due to the influence of temperature and humidity.

In this study, Kitchen smoke (chimney) is highly associated with risk of acute asthma exacerbation.

Those who have no kitchen smoke in their kitchen were 2.3 times at risk to develop acute asthma exacerbation [AOR = 2.307,95%CI = 1.010,5.2725] than those who have kitchen smoke. This finding is comparable with the study conducted in India [13]. This is due to the fact that kitchen smoke (chimney) is a way that helps in removing the smokes and fumes from the kitchen and making it clean and smoke free which result in reduction of indoor air pollution and prevents acute asthma exacerbation [23]. Inhaling harmful smoke can inflame lungs and airway, causing them to swell and block oxygen. This can lead to acute asthma exacerbation [24]

## Conclusion

In this study the determinant factors of acute asthma attack were spring season, presence of upper respiratory tract infection (URTI), having no Kitchen smoke in their kitchen and having obstructive sleep apnea.

### Limitations

The diagnosis of respiratory tract infections and sleep apnea was empirical (without laboratory) and all measures used were based on self-reporting, this might end up with social desirability bias. This study may have recall bias, since some of the information was based on the recall of the study participants. Unavailability of studies on acute asthma exacerbation.

## Supplementary information

**Additional file 1.** Annex I: English version structured interview questionnaire.

## Data Availability

The datasets used and analyzed during the current study are presented within the manuscript and available from the corresponding author on reasonable request.

## Abbreviations

AAA: Acute Asthma Attack
AOR: Adjusted Odds Ratio
CI: Confidence Interval
COR: Crude Odds Ratio
CSA: Central Statistical Agency
IQR: Interquartile Range
NHIS: National Health Interview Survey
OPD: Out Patient Department
TRHDA: Tigray Region Health Development Agency
URTI: Upper Respiratory Tract Infection
VIF: Variance Inflation Factor
