# Supplementary material for: Determinants of Acute Asthma Attack among adult asthmatic patients visiting hospitals of Tigray, Ethiopia, 2019: case control study
Source: Asthma Res Pract. 2020 Apr 3;6:1. doi: 10.1186/s40733-020-00054-w (PMC7118945; doi:10.1186/s40733-020-00054-w)
Supplement: Supplementary file 1 — Additional file 1. Annex I: English version structured interview questionnaire. [file 40733_2020_54_MOESM1_ESM.docx]

Annex I: English version structured interview questionnaire

Part I. Scio-demographic characteristics on determinant of acute asthma attack among adult patients visiting into three general hospitals of central zone, Tigray, Ethiopia, 2019

| **Part I. Scio-demographic characteristics** | | | | | |
| --- | --- | --- | --- | --- | --- |
| S. No | Questions | Alternative choice for respondents | | Skip to Q-- | |
| 1. 1 | Sex | Male … … ……………… … 1  Female … ……………… … ..2 | |  | |
|  | Age | _____ years | |  | |
|  | Religion | Orthodox … …………… … ...1  Muslim … ……………… … ..2  Protestant … …………… … ..3  Catholic … ……………… … .4  Others _______________________ | |  | |
|  | Ethnicity | Tigray … ……………… … .....1  Amhara … …………… … .. … .2  Oromo … ………………… … .3  Other specify______________ | |  | |
|  | Educational status | Able to read and write … … … 1  Unable to read and write … … .2  Primary school … … ……… … 3  Secondary school … ……… … .4  College and above … ……… … 5 | |  | |
|  | Occupational status | \| Government employed … … ..1 \| \| --- \| \| Self-employed/ Business … ...2  Unemployed … … ……… … ..3 \| | |  | |
|  | Marital status | Never married … ………… … ..1  Married … ………… … .. … … ..2  Divorced … ……………… … ..3  Widowed … ……………… … .4  Other … ………………… … ...5 | |  | |
|  | Place of residence | Urban … ………………… … ..1  Rural … ………………… … ..2 | |  | |
|  | Monthly income in ETB | <500 … ………………… … ...1  501-1500 … ……………… … 2  1501-2500 … …………… … ..3  >2500 … ………………… … .4 | |  | |
| **Part II: Behavioral factors** | | | | | |
|  | Have you ever smoked cigarettes? | Yes … … ………………… … ..1  No … ……………………… … 2 | | No, skip to  Q 204 | |
|  | Do you smoke cigarettes currently? | Yes … … ………………… … ..1  No … ……………………… … 2 | |  | |
|  | If no for Q 202 when do you stop? | <one year … …………… … . … 1  >one year … …………… … . … 2 | |  | |
|  | Is there anyone who smokes around you? | Yes … … ………………… … ..1  No … ……………………… … 2 | | No, skip to Q 206 | |
|  | If yes for Q 204, where? | At home … ………… … .. … … .1  At work place … … … `.............2  Other_____________________ | |  | |
|  | Do you do vigorous activity for at least 10 minutes continuously, that increases your breathing? like carrying or lifting heavy loads, digging or construction work, cutting fire wood for at least 10 minutes continuously? | Yes … … ………………… … ..1  No … ……………………… … 2 | |  | |
|  | Do you do moderate activity for at least 10 minutes continuously, that increases breathing? such as brisk walking or carrying light loads, washing clothes? | Yes … … ………………… … ..1  No … ……………………… … 2 | |  | |
|  | In the pass one week have you been engaged in any work related, transportation or recreational physical activities for >30min/day& for≥ 5 days/week? | Yes … … ………………… … ..1  No … ……………………… … 2 | |  | |
| **Part III: Environmental factor** | | | | | |
|  | Have you had an attack of asthma in the last 7 days? | Yes … ……… … … … … … 1  No … … ………………… … 2 | | No, skip to Q 303 | |
|  | If yes for Q 301  How many attacks? | ____________________ times | |  | |
|  | Is there any season that your attack becomes exaggerated? | Yes … ………………… … 1  No … … ……………… … 2 | | No, skip to Q 305 | |
|  | If yes for Q 303, at which season/month becomes most exaggerated? | Autumn .................................1  Winter … ………………… … 2  Spring … …… .. … …… . … … .3  Summer … ……… … .. … … … 4 | |  | |
|  | Have you ever had to leave your jobs because they affected your breathing? | Yes … …………………… … 1  No … …………………… … .2 | |  | |
|  | Have you been exposed to Vapors? | Yes … …………………… … 1  No … …………………… … .2 | |  | |
|  | Have you been exposed to Gases? | Yes … …………………… … 1  No … …………………… … .2 | |  | |
|  | Have you been exposed to Dust | Yes … …………………… … 1  No … …………………… … .2 | |  | |
|  | Have you been exposed to Fumes | Yes … …………………… … 1  No … …………………… … .2 | |  | |
|  | Have you been exposed to Humidity | Yes … …………………… … 1  No … …………………… … .2 | |  | |
|  | What do you mostly use for cooking?  Please select one | Coal/wood … … ………… … 1  Gas … ………………… … . .2  Electric … … ………… … … 3  Others___________________ | |  | |
|  | Do you open the door/windows while you were cooking? | Yes … …………… … . … … . … 1  No … ……………… … .. … … .2 | |  | |
|  | If yes for Q 312, How often? | Most of the time … ……… … ..1  Rarely … … ……………… … .2 | |  | |
|  | Did your kitchen have kitchen smoke? | Yes … …………………… … 1  No … …………………… … .2 | |  | |
| **PART IV: Medical and Clinical characteristics** | | | | | |
|  | Dose the participants have upper respiratory tract infection? | | Yes … … ……………… … .1  No … …………………… … 2 | |  |
|  | Dose the participants have obstructive sleep apnea? | Yes … … ……………… … .1  No … …………………… … 2 | |  | |
|  | Have you ever missing follow up? | Yes … … ………… … . … .1  No … …………… … . … … 2 | |  | |
|  | With In the six months for how many do you missing your follow up? | One time … … …………… … ..1  Two times … … …………… … 2  Three times … … …… … .. … … 3 | |  | |
